# Laser-based selective killing of a manipulative parasite reveals partial reversibility of phenotypic alterations in its intermediate host

**DOI:** 10.1016/j.crpvbd.2024.100221

**Published:** 2024-10-11

**Authors:** Marie-Jeanne Perrot-Minnot, Frank Cézilly, Olivier Musset

**Affiliations:** a Biogéosciences, UMR 6282 CNRS Université de Bourgogne, 6 Boulevard Gabriel, 21000, Dijon, France; b Université de Bourgogne, 6 Boulevard Gabriel, 21000, Dijon, France; c Laboratoire Interdisciplinaire Carnot de Bourgogne, UMR 6303 CNRS, Université Bourgogne Franche-Comté, 9 Avenue Alain Savary, 21078, Dijon, France

**Keywords:** Complex life-cycle, Costs, Crustacea, Immunity, Recovery

## Abstract

Various parasites alter their intermediate hostʼs phenotype in ways that increase parasite transmission to definitive hosts. To what extent infected intermediate hosts can recover from such “manipulation” is poorly documented, thus limiting our understanding of its proximate and ultimate causes. Here, we address the reversibility of several phenotypic alterations induced by the acanthocephalan *Polymorphus minutus*, a trophically-transmitted bird parasite, in its amphipod intermediate host. Using a recently developed laser-based technology, we selectively killed parasite larvae inside the body cavity of *Gammarus fossarum*, while preserving host viability. Following behavioral tests, parasite death was confirmed using DNA integrity assays. Alterations of geotaxis, locomotor activity and resting metabolic rate in infected gammarids remained unchanged one month after parasiteʼs death. In contrast, elevated brain lactate concentration and hemolymph total phenoloxidase activity of treated gammarids hosting a dead cystacanth returned to control (uninfected) levels. Interestingly, melanotic encapsulation response to dead cystacanths was rare up to two months after treatment, with only 5.6% of cystacanths being fully or partially melanized, thus suggesting long-lasting protection from the acellular outer envelope. Irreversible behavioral but reversible physiological alterations appear to be a cost-effective strategy of host manipulation, and point to a putative role of epigenetic alterations in parasite manipulation.

## Highlights

• Laser-based selective killing is used to assess the reversibility of host manipulation by parasites.
• Negative geotaxis induced by a bird acanthocephalan in its intermediate host is irreversible.
• Alterations of brain lactate concentration and humoral immunity are reversible.
• Lack of encapsulation of dead parasites suggests a masquerading effect.
• Irreversible behavioral but reversible physiological alterations is a cost-effective strategy.

## Graphical abstract

**Figure:**
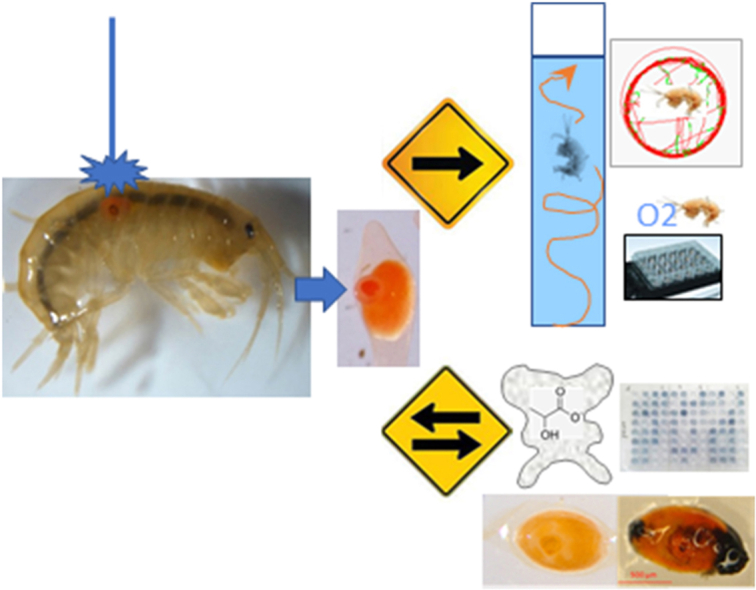

## 1 Introduction

Several parasites with complex life-cycles can induce various phenotypic alterations to their intermediate hosts that appear to increase trophic transmission to definitive hosts (Poulin, 1995; Moore, 2002; Lefèvre et al., 2009; Cézilly et al., 2010; Poulin, 2010; Hughes et al., 2012; Lafferty and Shaw, 2013; Iritani and Sato, 2018; Bhattarai et al., 2021; Tong et al., 2021). Some parasite-induced phenotypic alterations can be quite conspicuous, as in the case of modified host appearance or behavior, or more subtle in the case of some physiological changes (Poulin, 2010; Bakker et al., 2017; Fayard et al., 2020). The purposive design of behavioral alterations (Poulin, 1995; Thomas et al., 2005; Bakker et al., 2017; Bhattarai et al., 2021) and experiments evidencing their role in enhanced trophic transmission (Lafferty and Morris, 1996; Lagrue et al., 2007; Perrot-Minnot et al., 2007; Dianne et al., 2011; Jacquin et al., 2014) have been largely regarded as strong support for the hypothesis of adaptive manipulation of hosts by parasites (see however Cézilly et al., 2010).

Despite a growing interest in how these parasites take control of their hosts (Adamo, 2002; Poulin, 2010), our understanding of the involved physiological and molecular mechanisms remains limited (Herbison, 2017; Herbison et al., 2018). In particular, little is known about whether phenotypic alterations are the consequences of a continuous cross-talk between the parasite and its hostʼs neuroendocrine-immune network and, hence, might be reversible, or whether they consist in permanent, structural alterations that are irreversible. The question is of importance from both an ultimate and a proximate point of view. From the parasiteʼs point of view, adaptive manipulation is expected to come with physiological costs, as discussed theoretically (Thomas et al., 2005; Parker et al., 2009; Poulin, 2010; Vickery and Poulin, 2010) but not evidenced experimentally yet (Hafer-Hahmann, 2019). However, such costs must be balanced against the risk of dying inside the intermediate host before transmission to a definitive host occurs, or that of predation by an unsuitable definitive host (Parker et al., 2009; Poulin, 2010; Vickery and Poulin, 2010). In terms of adaptive consequences, irreversible manipulation could therefore reflect a cost-effective strategy of behavioral manipulation, by which parasites reduce the physiological costs of manipulation without trading off transmission efficiency. From the intermediate hostʼs point of view, the possibility to recover from parasitic manipulation should favor continuous resistance to the parasite, and eventually lead to reversible phenotypic alterations. Parasites could mitigate the physiological cost of manipulation induction using either one of two alternative strategies. On the one hand, they could continuously secrete molecular effectors at a relatively low energetic cost to fight against host resistance. On the other hand, they could induce large and long-lasting structural or chemical alterations at once (Adamo, 2013), incurring a higher but temporary energetic cost. The former strategy would imply reversible phenotypic changes upon the breaking down of host-parasite continuous cross-talk, while the latter would result in irreversible parasite-induced phenotypic alterations.

The reversibility of manipulation by parasites with complex life-cycles has not been examined so far despite its direct relevance to the ongoing debate about the adaptive nature of intermediate host manipulation by parasites (Thomas et al., 2005; Cézilly et al., 2010). This is mainly explained by the difficulty to neutralize parasites within their hosts, and more specifically helminth macroparasites in their invertebrate intermediate hosts. This is particularly true of acanthocephalan parasites and their crustacean intermediate hosts, a favorite system in the study of host-manipulation by parasites (Bakker et al., 2017; Fayard et al., 2020; Perrot-Minnot et al., 2023). Acanthocephalans can induce multiple phenotypic alterations in their hosts (Cézilly and Perrot-Minnot, 2010; Fayard et al., 2020) that contribute to a certain extent to enhanced transmission to definitive hosts, as evidenced in both field (Lagrue et al., 2007; Perrot-Minnot et al., 2007) and laboratory experiments (Perrot-Minnot et al., 2007, 2012; Dianne et al., 2011; Jacquin et al., 2014). However, the proximate mechanisms behind phenotypic manipulation are still poorly documented and restricted to a few candidate physiological pathways (Perrot-Minnot et al., 2023).

Among acanthocephalans, *Polymorphus minutus* is particularly suited to address the reversibility of manipulation. This parasite is known to induce several phenotypic alterations to its crustacean amphipod hosts, including reversed geotaxis (Perrot-Minnot et al., 2016 and references therein), modified locomotor activity (Marriott et al., 1989; Thünken et al., 2010; Jacquin et al., 2014; Rothe et al., 2022), altered metabolic rate and brain lactate level (Perrot-Minnot et al., 2016), and immunosuppression (Cornet et al., 2009). Some of these alterations are expected to increase parasite transmission success, either by increasing predation rate by definitive hosts (Jacquin et al., 2014) or by facilitating parasite development and survival through energy reallocation or lowered immune defenses. At the proximate level, the mechanistic causes possibly accounting for the phenotypic alterations induced by *P. minutus* have not been unraveled yet, as for acanthocephalans in general (Perrot-Minnot et al., 2023). Since behavioral modulation is orchestrated in the central nervous system, studies investigating proximate mechanisms of manipulation have been focusing on brain neurochemistry, although acanthocephalans “act” at a distance from the body cavity of their intermediate host through unknown excretion-secretion products (Perrot-Minnot et al., 2023). Pioneer proteomic analysis on the brain of *P. minutus*-infected gammarids has revealed the differential expression of proteins involved in the central nervous system development and in the immune system (Ponton et al., 2006). Using a candidate-system approach to address the physiological basis of altered geotaxis, Perrot-Minnot et al. (2016) provided evidence for the implication of brain lactate in the modulation of geotaxis, and for increased lactate concentration in the brain of *P. minutus*-infected *Gammarus roeseli*.

Here, we took advantage of a newly-developed laser-based technique allowing specific killing of *P. minutus* cystacanths without affecting its intermediate host, the freshwater amphipod *Gammarus fossarum* (Musset et al., 2023). We were thus able to compare geotaxis, locomotor activity, metabolic rate, and immune defense between gammarids hosting a dead cystacanth and both gammarids harboring a live cystacanth and uninfected gammarids. We also assessed immunocompetence from one key component of arthropod innate immune defense, the phenoloxidase system. The prophenoloxidase system (total ProPO and PO activity, thereafter ProPO-PO) was expected to be depressed in the hemolymph of gammarids infected with *P. minutus* cystacanths compared to uninfected ones (Cornet et al., 2009). In addition, reversible immunosuppression should reactivate the phenoloxidase cascade, thus allowing the melanotic encapsulation of the dead cystacanth*.* Finally, we compared brain lactate concentration according to infection status and parasite viability, in an attempt to address the proximate mechanism of geotaxis alteration and its reversibility. We addressed the reversibility of these behavioral and physiological alterations one to two months after laser-based killing, a duration long enough to detect reversion given acanthocephalan developmental time and gammarid lifespan. If host manipulation relies on a continuous cross-talk between *P. minutus* and its host (neuro)physiological systems, we expected to observe altered traits returning to their original levels (i.e. as observed in uninfected gammarids) among gammarids hosting a dead cystacanth. Alternatively, if irreversibility of manipulation by *P. minutus* has been selected and infection induces permanent structural or signaling alterations, we expected that infected gammarids would not recover from *P. minutus-*induced alterations following parasite death.

## 2 Materials and methods

### 2.1 Amphipod collection and maintenance

Gammarids were collected by kick-sampling in the river Bèze at Noiron sur Bère (Burgundy, France; Lat. 47.437058, Long. 5.304646) during springtime over three consecutive years. They were allowed to acclimate in the laboratory for at least one week prior to the onset of experiments. During this time, they were maintained in tanks filled with oxygenated dechlorinated ultraviolet (UV)-treated tap water (hereafter TW) mixed with water and rocky substrate from the river in a temperature- and photoperiod-controlled room (16 °C, 12 L:12 D, 800 Lux illumination). Gammarids were fed weekly on decaying elm leaves and chironomid larvae.

### 2.2 Laser treatment and assessment of parasiteʼs death

Cystacanths of *P. minutus* were exposed to the deleterious thermal effects of laser irradiation through the host cuticle, following Musset et al. (2023). Briefly, individual gammarids were anesthetized for 20–30 min in a MS222 anesthetic bath, and then positioned on a cooled sample holder for exposure to a Blue Laser Diode irradiation at 450 nm. The laser treatment consisted in pre-drilling a hole in the cuticle at 0.5 W for 100 ms, before applying five pulses of 50 ms at 1.4 W, interspaced by 100 ms. For long-term maintenance (one to two months), gammarids were pooled by status - not infected, infected controls not exposed to laser treatment, and infected exposed to laser treatment - and returned to separate large tanks under the same conditions as above. We distinguished live from dead parasites one or two months after laser treatment, using DNA integrity assay, following Musset et al. (2023). Briefly, we assessed the integrity of total DNA from deep-frozen cystacanth after restricted digestion, concentration by freeze-dried lyophilization, and visualization of either intact high molecular weight DNA or degraded DNA smear with agarose gel electrophoresis.

**Fig. 1:**
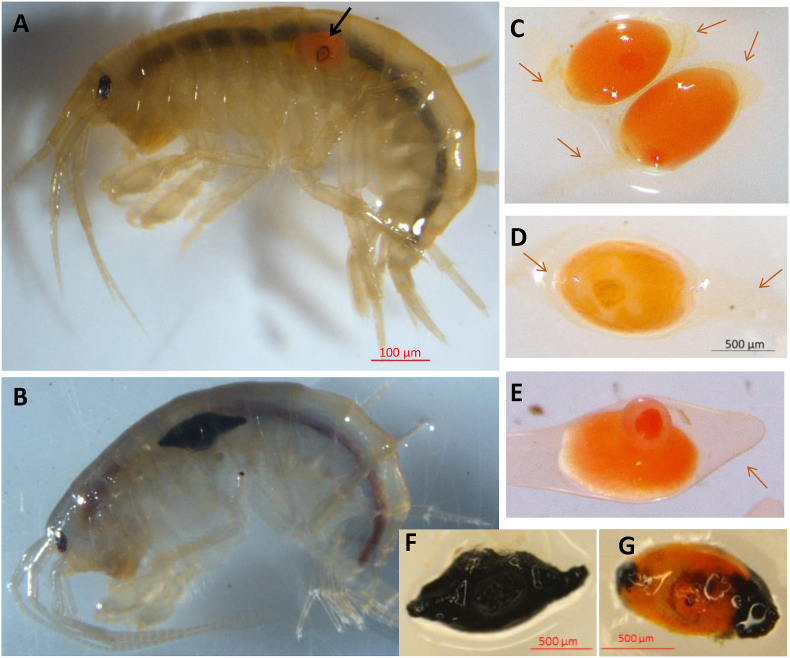
Appearance of *Polymorphus minutus* cystacanths inside (**A**, **B**) or dissected out its host, *Gammarus fossarum* (**C**–**G**) following exposure to Blue Diode Laser beam within live host. The bright orange color of the cystacanth makes it clearly visible through its hotsʼs translucid cuticle, allowing a precise positioning of the laser beam, as shown at the impact point (*black arrow*) few hours after exposure (**A**). On exceptional cases, laser-killed cystacanth showing full melanization was visible through the cuticle of live host (**B**) or dissected out (**G**) two months after laser exposure. Most of dead parasites were harboring a visible wound of varying appearance (83%; **C** to **G**). Only 5.6% of exposed cystacanths were found fully or partially melanized two months after laser treatment (**F**, **G**); 13.5% had few melanization spots on the outer envelope in addition to the wound, half of them being stunted (not shown). In most others, the wound looked like a small to medium (62.9%; **C**) or large (6.7%, **D**, **E**) protrusion with occasional bleaching at the point of injury (**D**), and a few dead cystacanth did not exhibit any visible wound (11.2%). Arrows indicate the outer envelope, visible and intact in all cystacanths exposed to laser irradiation (except the partially to fully melanized ones).

Exposition to laser irradiation left most infected gammarid hosts with little if any damage, as the targeted parasite absorbed most if not all the energy delivered (be it surviving to it or not) (Musset et al., 2023). Conversely, uninfected gammarids exposed to laser beam or infected gammarids exposed to laser treatment without targeting the cystacanth systematically experienced severe physiological damage, as all the energy delivered propagated to the host tissues and induced lethal/sublethal effects (Musset et al., 2023). Therefore, it was not possible to use uninfected gammarids exposed to laser treatment as conventional controls. However, on some occasions, the exposed cystacanth in infected gammarids did survive laser treatment. We then used gammarids harbouring a “survivor” cystacanth as our control group to test for potential confounding effects associated with the laser treatment in itself. We could thereby address manipulation reversibility while controlling for laser treatment, by comparing gammarids infected with a “survivor” *P. minutus* cystacanth (26 out of 110) to gammarids hosting a live cystacanth unexposed to laser irradiation (effect of laser alone), and gammarids hosting a laser-killed cystacanth (effect of parasite death alone). These comparisons were run in a separate analysis.

### 2.3 Geotaxis, metabolic rate, and locomotor activity

We conducted behavioral tests one month and two months after laser treatment, except for locomotor activity (one month). We performed behavioral assays in the temperature- and photoperiod-controlled room where gammarids were maintained. We assessed geotaxis from the position of an individual gammarid in a cylindrical plastic column filled with water, and divided into five zones of equal size from the bottom to the top (Perrot-Minnot et al., 2014). The inner walls of the columns were covered with plastic netting to offer a substrate to cling on, as available on riverbanks. Geotaxis was scored under low and homogeneous illumination (200 Lux) to control for phototaxis. After an acclimatization time of 3 min, the position of the gammarid along the water column was recorded every 30 s for 10 min and scored from 1 (bottom) to 5 (top compartment). The cumulated geotaxis score therefore ranged from 20 to 100. In addition, we assessed the repeatability of geotaxis at a 24-h interval on a subset of individuals. We quantified locomotor activity on a subset of gammarids one month after laser treatment, by recording the proportion of time spent swimming or being immobile using a real-time automated recording device (ZebraLab, ViewPoint ®, Lyon, France), following Perrot-Minnot et al. (2014), with modifications. Following a 2-min period of acclimatization, we video-recorded the locomotor activity of individual gammarids in a Petri dish filled with TW under moderate light intensity (400 Lux) during 3 min. Based on preliminary tests, we considered time spent motionless (movement speed < 7 mm/s), time spent in crawling activity (movement speed between 7 and 15 mm/s) and time spent in swimming activity (speed above 15 mm/s).

Following Perrot-Minnot et al. (2014), we assessed resting metabolic rate (RMR) by quantifying oxygen consumption rate using optical fluorescence-based oxygen respirometry and the SensorDish device (SDRv4, Presents, Regensburg, Germany; batch OD-1333-01 calibrated at 16 °C). To estimate individual RMR, we subtracted the mean value of control wells to that of experimental wells in order to get the amount of oxygen consumed by individual gammarids. We then derived the RMR (in micrograms O_2_/min) from the slope of the linear regression of this parameter on time. Since the size of the wells allowed limited movement by gammarids, our measure of metabolic rate slightly overestimates RMR.

### 2.4 Hemolymph ProPO-PO and protein concentration, and brain lactate level

Gammarids were dissected 24–48 h after the last behavioral test. Gammarids were rapidly decapitated with forceps on an ice-filled Petri dish and kept on the lid for hemolymph collection (1–2 μl) and brain dissection (after carefully casting off the cephalic skeleton). Pools of three brains and of hemolymph from three gammarids were readily deep frozen in LN2 and stored at −80 °C. Brain and hemolymph were collected from 11:00 to 16:30 h to limit the putative impact of circadian fluctuations on brain chemistry. We then dried the body remains in an incubator for at least 3 days at 40 °C and weighed them to the nearest 0.01 mg with an analytical balance (Precisa 262SMA-FR, Precisa Instruments, Bisingen, Switzerland).

The phenoloxidase system comprises both the pro-phenoloxidase (ProPO) and phenoloxidase (PO), PO being the activated form of ProPO circulating in the hemolymph and is involved in the melanotic encapsulation of foreign body by invertebrates (González-Santoyo and Córdoba-Aguilar, 2012). We quantified and compared pro-phenoloxidase (ProPO) and phenoloxidase (PO) total activity according to infection and parasite viability, and time since laser treatment (one or two months). We also assessed the investment in total ProPO-PO activity relative to total protein concentration in the hemolymph. Three microliters of pure hemolymph were mixed with 17 μl of ice-cold PBS pH 7.4 in microplate wells. Phenoloxidase (PO) and total activity (ProPO-PO) enzymatic assay was immediately performed according to Cornet et al. (2009) using 5 μl of diluted hemolymph for each. We quantified enzyme activity as the slope of the curve corresponding to the linear phase of the reaction (Vmax-value) adjusted to the activity of 1 μl of pure hemolymph. We quantified total protein concentration in 2 μl of diluted hemolymph using the Bradford method (DC™ Protein Assay kit (BioRad)) and Bovin Serum Albumin (BSA) as standard (0.48–0.046 μg/μl) following the manufacturerʼs instructions, expressed in micrograms per microliter of pure hemolymph. To complement the quantification of PPO-PO activity, we assessed the extent of melanotic encapsulation of an inorganic and non-pathogenic foreign body. We implanted nylon monofilament into the body cavity of another set of infected gammarids exposed to laser treatment, unexposed infected gammarids, and uninfected gammarids, six weeks after laser treatment. After six days, we retrieved implants and quantified the amount of melanic pigments deposited using Image J software (see detailed protocol in Supplementary material S5).

We estimated brain lactate level two months after treatment using enzymatic assay and spectrophotometric determination (enzymatic ultraviolet L-lactate kit K-LATE, Megazyme Intl, Wicklow, Ireland) following Perrot-Minnot et al. (2016) with slight modifications. Briefly, homogenization and deproteinization of brain tissue were done in 10 μl ice-cold 1M PCA in a single step, by sonication for two 90-s rounds in an ultrasonic bath with ice-cold water, interspaced with short centrifugation at 4 °C and two 1-min rounds grinding with few glass beads using a ball mill RETSCH MM 400 Mixer Mill, and a few min on ice. After 10 min in PCA and the addition of 10 μl of ice-cold 1M KOH, samples were thoroughly vortexed and then centrifuged at 6000 rpm at 4 °C for 3 min. Eighteen microliters of clear supernatant were collected in an ice-cold microplate, and the lactate assay was run immediately after following the manufacturerʼs instructions. Lactate concentration per brain was estimated from the optical density measured at 30 min using lactate as standard (0.15–0.004 μg/μl).

All dosages were conducted using microplate spectrophotometers SpectraMax® Plus384 and iD3 (Molecular Devices LLC, Sunnyvale, CA, USA). Samples from the three groups were randomized on each microplate assay. Because we had to pool hemolymph and brains during dissection before we could conduct DNA-integrity assay for parasite death, we had to subsequently discard several samples mixing hemolymph or brain from gammarids hosting a dead cystacanth and a live one (not killed by laser treatment).

### 2.5 Statistical analysis

We analyzed the effect of infection and parasite viability on geotaxis by performing a Kruskal-Wallis test on geotaxis score, followed by Dunnʼs test for multiple comparisons with Benjamini-Yekuteili correction (R-package “*dunn test*”, Dinno, 2017). We performed generalized linear regression models (GLM) to analyze locomotor activity, metabolic rate, and total ProPO-PO activity and protein concentration in hemolymph (R-package “*lme4*”, Bates et al., 2015). The choice of variance and link functions was made based on prior visualization of the distribution of the dependent variable, and adjusted based on residual plots visualization. For model reduction, we used a likelihood ratio test to compare the relative goodness-of-fit of nested models (R-package “*lmtest*”, Zeileis and Hothorn, 2002). We run an analysis of deviance on the reduced model and tested the effect of each predictor using the sums of squares for each predictor conditional on the other predictors and associated *P*-values (type II tests) (R-package “*car*”, Fox and Weisberg, 2019). *Post-hoc* paired comparison between groups were performed using Tukey adjustment for multiple comparisons (R-package “*emmeans*”, Lenth, 2023). We reported the McFadden *R*-squared as an estimate of goodness of fit. We analyzed the effect of infection and parasite viability on locomotor activity by fitting a GLM to the proportion of time spent immobile (speed below 7 mm/s) or swimming (speed above 15 mm/s), using the quasibinomial family function.

We analyzed the effect of infection and parasite viability on RMR (oxygen consumption per unit of time) by fitting a GLM to resting metabolic rate using Gaussian family and log-link function, with infection and parasite viability, log_10_-transformed dry body weight, and time since laser treatment (one or two months), as explanatory variables. Dry body weight was incorporated as a predictor, following the allometric metabolic scaling rule whereby metabolic rate is expected to increase with gammarid weight (Glazier, 2005). Gammarid dry weight according to gammarid groups and time since laser treatment was also analyzed using a generalized linear model with Gaussian family and log-link function. We analyzed differences in hemolymph phenoloxidase activity by fitting a GLM with Gaussian family and square root-link function, using infection and parasite viability, total protein concentration in hemolymph, and time since laser treatment (one or two months) as predictors. Hemolymph total protein according to gammarid group and time since laser treatment was also analyzed using a generalized linear model with Gaussian family and log-link function. The extent of melanotic encapsulation was assessed from the density of melanization, and its variation according to experimental group was analyzed using linear model (Supplementary material S5).

Due to low sample size (*n* = 11–19) and departure from normality, lactate concentration in pools of three brains (occasionally two) was analyzed using non-parametric tests. We ran a Kruskall-Wallis test, followed by *post-hoc* paired Dunnʼs tests and accounted for multiple paired comparisons using the Benjamini-Yekuteili correction (R-package “*dunn test*”, Dinno, 2017).

All analyses were done on R v. 4.3.1 (R Core Team, 2023) in RStudio 2023.06.2 (Posit Teams, 2023), and results were considered significant at the 0.05 level.

## 3 Results

Among the 110 *P. minutus* exposed to laser treatment, 84 were diagnosed as dead and 26 were diagnosed as alive based on DNA degradation and integrity, respectively (mortality rate of parasites due to laser treatment = 76.4%). The proportion of dead parasites did not significantly differ between one month and two months after laser treatment (33 out of 41 *vs* 51 out of 69, respectively; Chi-square test, *χ*^2^ = 0.62, *P* = 0.43). Only two parasites were melanized, one fully and another one partly (Fig. 1F and G). Most cystacanths exhibited a small to large protruding wound (Fig. 1C–E), and a few exhibited small melanin dots on the intact outer envelope in addition to the wound.

### 3.1 Geotaxis, locomotor activity and metabolic rate are irreversibly altered by *P. minutus*

**Fig. 2:**
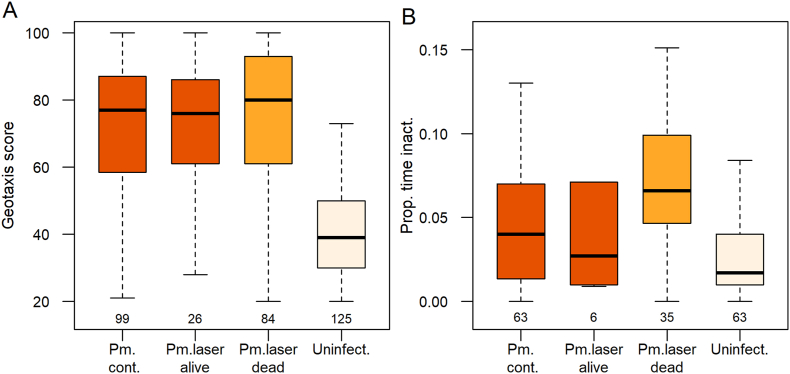
Behavior of individual *Gammarus fossarum* infected with *Polymorphus minutus* or uninfected, recorded one to two months after selective laser-based killing of the parasite. **A** Geotaxis. **B** Locomotor activity, recorded as the proportion of time spent immobile (< 7 mm/s). Gammarids were infected with either a live cystacanth not exposed to laser irradiation (Pm. cont.), a cystacanth having survived laser treatment (Pm. laser alive), a dead cystacanth of *P. minutus* (Pm.dead), or were uninfected (Uninfect.). Sample size is given below boxplots.

Geotaxis scores differed significantly among gammarid groups (*χ*^2^ = 134.59, *df* = 2, *P* < 0.0001; Fig. 2A), and was significantly repeatable within each group (Supplementary material S1). Geotaxis of uninfected gammarids was significantly altered from positive to negative in infected gammarids, including after selective killing of the parasite (Table 1, Fig. 2). There was no difference in geotaxis within each group according to whether it was measured one or two months after laser treatment (Supplementary Fig. S1).

**Table 1:** Effect of infection of the amphipod *Gammarus fossarum* with live or dead *Polymorphus minutus* on geotaxis, locomotor activity, metabolic rate, immunity and brain lactate. *Post-hoc* paired comparisons were run between gammarids infected with live *P. minutus* unexposed to laser treatment (infected controls), infected with laser-killed *P. minutus* (dead), and uninfected gammarids, according to the predictions from the reversibility hypothesis.

	Dead *vs* live *P. minutus*	Dead *P. minutus vs* uninfected	Live *P. minutus vs* uninfected
Reversibility hypothesis:	Significant (recovery)	Non-significant (recovery)	Significant (manipulation)
Behavior			
Geotaxis	*Z* = −0,87, *P = *0.35	*Z* = 10.01, *P* < 0.0001^a^	*Z* = 9.54, *P* < 0.0001^a^
Locomotor activity			
‣ Immobility	*Z* = −1.96, *P* = 0.13	*Z* = 4.18, *P* = 0.0001^a^	*Z* = 2.59, *P* = 0.026^a^
‣ Swimming	*Z* = −3.10, *P* = 0.006^b^	*Z* = −5.07, *P* < 0.0001^a^	*Z* = −2.30, *P* = 0.055
Physiology			
‣ Resting metabolic rate at one month	*t* = −0,10, *P* = 0.78	*t* = −3.301, *P* = 0.003^a^	*t* = −4.95, *P* < 0.0001^a^
‣ Immunity (total phenoloxidase system: ProPO-PO)	*t* = −2.47, *P* = 0.044^a^	*t* = 0.52, *P* = 0.86	*t* = 3.22, *P* = 0.006^a^
‣ Brain lactate concentration	*P = *0.05^a^	*P* = 0.73	*P = *0.03^a^

a Values are statistically significant and can be contrasted to the hypothesis of reversibility of parasite-induced alteration.

b Values are statistically significant but at the opposite of expectations based on the reversibility hypothesis.

Locomotor activity differed significantly among gammarid groups (LR Chi-square test: immobility: *χ*^2^ = 18.10, *df* = 2, *P* = 0.0001, *R*^2^ = 0.12; swimming activity: *χ*^2^ = 25.87, *df* = 2, *P* < 0.0001, *R*^2^ = 0.13). The proportion of time spent immobile increased whereas the proportion of time spent swimming decreased in gammarids infected with a dead cystacanth, compared to uninfected ones (Table 1, Fig. 2B, Supplementary Fig. S3). Locomotor activity also decreased, but to lower extent, in infected control gammarids compared to uninfected ones (Table 1, Supplementary Fig. S3). Gammarids hosting a dead parasite spent less time swimming compared to those hosting a live parasite (Table 1, Supplementary Fig. S3), moving more often by crawling at low speed (Supplementary Fig. S3).

**Fig. 3:**
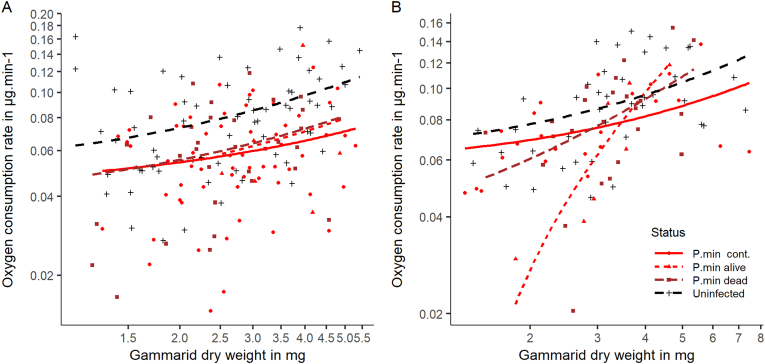
Metabolic rate of *Gammarus fossarum* infected with *Polymorphus minutus* or uninfected, recorded one month (**A**) or two months (**B**) after laser-based killing of the parasite using a Blue Diode Laser beam. Gammarids were infected with either a dead (laser-killed) *P. minutus* cystacanth (P.min dead: *brown dotted line*, *square*, N_a_ = 33 and N_b_ = 15), a live cystacanth not exposed to laser irradiation (P.min cont.: *red plain line*, *dot*, N_a_ = 63 and N_b_ = 24) or having survived laser treatment (P.min alive: *red dotted line*, N_a_ = 6 and N_b_ = 7), or were uninfected (*black dashed line*, *cross*, N_a_ = 64 and N_b_ = 41). Resting metabolic rate is reported in mg O_2_ consumed per min and is regressed on gammarid dry body weight (in mg) using a log-scale, following a relationship known as metabolic scaling.

Resting metabolic rate differed according to gammarid group, dry body weight, and time since treatment (*χ*^2^ = 73.6, *df* = 4, *P* < 0.0001, *R*^2^ = 0.27; Fig. 3). In addition, dry body weight differed according to time since treatment, but not according to gammarid group (Supplementary material S2). Therefore, we run separate analyses for the two post-treatment durations. After one month, RMR differed according to gammarid group and body weight (*χ*^2^ = 40.98, *df* = 3, *P* < 0.0001, *R*^2^ = 0.26; group: *χ*^2^ = 27.18, *df* = 2, *P* < 0.0001; weight: *χ*^2^ = 17.8, *df* = 1, *P* < 0.0001). RMR increased with gammarid dry weight and was lower in infected gammarids compared to uninfected ones, independently of parasite viability (Table 1, Fig. 3A). However, only dry body weight accounted for differences in RMR two months after treatment (*χ*^2^ = 27.6, *df* = 4, *P* < 0.0001, *R*^2^ = 0.27; group: *χ*^2^ = 3.99, *df* = 3, *P =* 0.26; weight: *χ*^2^ = 22.89, *df* = 1, *P* < 0.0001; Fig. 3B). RMR was significantly higher at two months compared to one month, in gammarids infected with either a live (*χ*^2^ = 9.82, *df* = 1, *P* = 0.002, *R*^2^ = 0.21) or a dead parasite (*χ*^2^ = 5.89, *df* = 1, *P* = 0.0015, *R*^2^ = 0.29), whereas the difference was no significant in uninfected ones (*χ*^2^ = 0.022, *df* = 1, *P* = 0.88, *R*^2^ = 0.17). The overall variance in RMR was much lower after two months compared to one month (Fig. 3).

The geotaxis and RMR of gammarids infected with a parasite having survived laser exposure did not differ significantly from unexposed infected gammarids and gammarids hosting a dead parasite (geotaxis, *χ*^2^ = 2.33, *df* = 2, *P* = 0.33; status effect on RMR at one month post-exposure, *χ*^2^ = 0.61, *P* = 0.74, at two months post-exposure, *χ*^2^ = 0.9, *P* = 0.64, respectively) (Fig. 2, Fig. 3). Locomotor activity was significantly different among these three groups of infected gammarids (immobility: *χ*^2^ = 6.19, *df* = 2, *P* = 0.05; swimming: *χ*^2^ = 9.11, *df* = 2, *P* = 0.01; Fig. 2A and Supplementary Fig. S3), as expected from the increased immobility of gammarids infected with a dead parasite (see above). The locomotor activity of gammarids infected with a parasite surviving laser exposure was comparable to that of unexposed infected gammarids, but not significantly different from that of gammarids hosting a dead parasite, possibly due to the relatively low sample size (Fig. 2B). Thereby, laser exposure appeared to induce negligible side-effect on the behavior of the host itself.

### 3.2 Hemolymph total phenoloxidase activity and brain lactate concentration returned back to levels of uninfected gammarids following parasiteʼs death

**Fig. 4:**
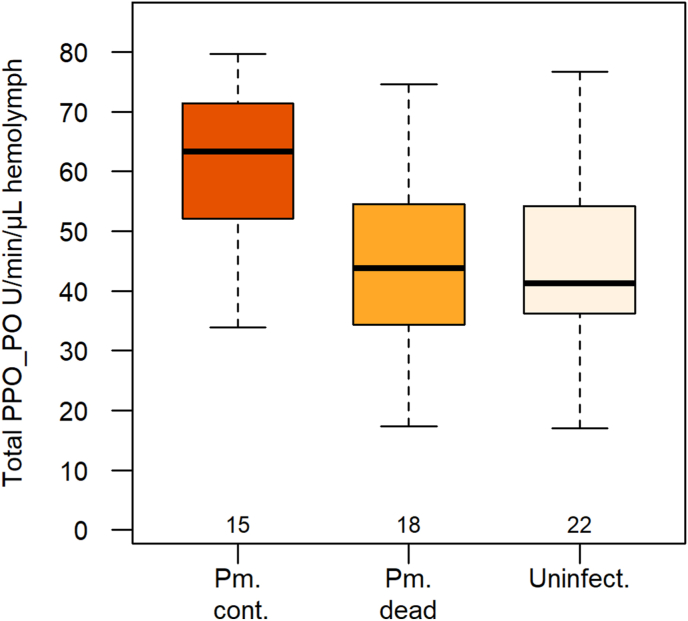
Phenoloxidase system as part of humoral immunity in the hemolymph of *Gammarus fossarum* infected with either a live cystacanth not exposed to laser irradiation (Pm. cont.) or a dead (laser-killed) cystacanth (Pm. dead), or were uninfected (Uninfect.). Total phenoloxidase activity (ProPO-PO) was quantified as Vmax, the maximum linear rate of substrate conversion in Units per minute per microliter of hemolymph (sample size given below boxplots).

Variation in hemolymph total phenoloxidase activity (ProPO-PO) was explained by gammarid group and hemolymph protein concentration, independently of time since treatment and interactions (reduced model: *χ*^2^ = 19.4, *df* = 3, *P* = 0.0002, *R*^2^ = 0.30; group: *χ*^2^ = 10.91, *df* = 2, *P* = 0.004; protein concentration: *χ*^2^ = 7.53, *df* = 1, *P* = 0.006; Supplementary Fig. S4A). The activity of ProPO-PO in gammarids hosting a dead cystacanth was comparable to that of uninfected gammarids, and lower than that of gammarids infected with a live cystacanth (Table 1, Fig. 4, Supplementary Fig. S4A). Gammarids infected with a live cystacanth had higher ProPO-PO activity than uninfected gammarids (Table 1, Fig. 4). Total protein concentration in hemolymph did not differ according to group, time since treatment, and their interaction (*χ*^2^ = 4.62, *df* = 5, *P* = 0.46, *R*^2^ = 0.02; Supplementary Fig. S4B). Finally, the extent of melanotic encapsulation of nylon implant was comparable among gammarid groups six weeks after treatment, as assessed in a complementary experiment (Supplementary Fig. S5). All gammarids hosting a dead cystacanth successfully melanized nylon implant (*n* = 7). This contrasts with the proportion of gammarids hosting a dead cystacanth, having fully or partly melanized the cystacanth (17 out of 86: Fisherʼs exact test, *P* < 0.0001). The analysis of immunity (PPO and encapsulation) was not performed on gammarids hosting a “survivor” parasite, due to too low sample size (*n* = 3) compared to the other groups.

**Fig. 5:**
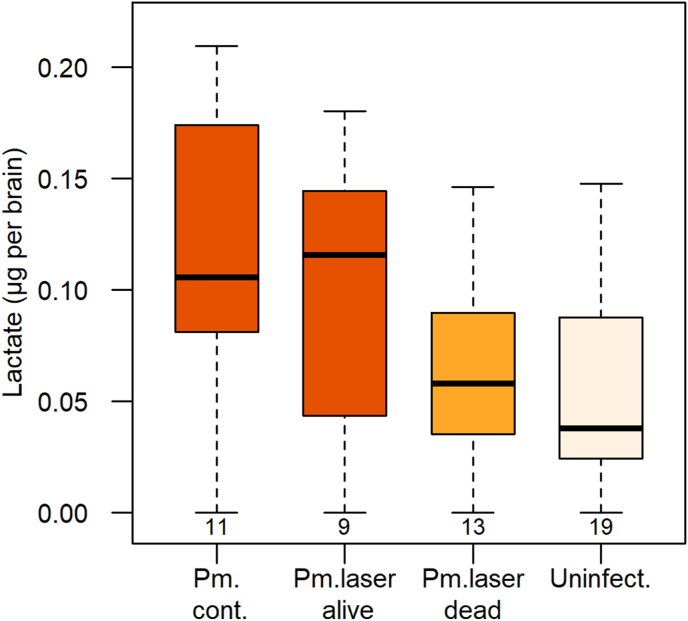
Variation in lactate concentration in the brain of *Gammarus fossarum* according to *P. minutus*-infection status and treatment: gammarids were infected with either a live cystacanth not exposed to laser irradiation (Pm. cont.) or having survived to laser treatment (Pm.laser alive), or with a dead (laser-killed) cystacanth (Pm. dead), or were uninfected (Uninfect.). Sample size is given below boxplots.

There was a significant tendency for lactate level to vary according to infection status and treatment (Kruskal-Wallis test, *χ*^2^ = 6.92, *df* = 2, *P* = 0.03; Fig. 5). *Post-hoc* tests showed that brain lactate concentration did not differ between gammarids infected with a dead cystacanth and uninfected ones, whereas both groups had significantly lower concentration of brain lactate compared to infected controls (Table 1, Fig. 5). In addition, lactate level in the brain of gammarids hosting a cystacanth having survived to laser treatment was comparable to that of unexposed infected gammarids, and was higher than that of gammarids hosting a dead parasite, albeit not significantly (Fig. 5; Kruskal-Wallis test on the three groups of infected gammarids, *χ*^2^ = 4.79, *df* = 2, *P* = 0.09).

## 4 Discussion

Our results provide new and original insights on the reversibility of parasite-induced phenotypic alterations. Negative geotaxis, one of the main behavioral alterations contributing to increased trophic transmission of *P. minutus* (Jacquin et al., 2014), was found irreversible one and two months after selective laser-based killing of the cystacanth. The same was true of reduced locomotor activity and decreased metabolic rate one month after treatment, two additional phenotypic alterations brought about by *P. minutus* in its amphipod host (Thünken et al., 2010; Jacquin et al., 2014; Perrot-Minnot et al., 2016). By contrast, both the humoral (hemolymph phenoloxidase concentration) and cellular (encapsulation response) immune responses, as well as brain lactate level, were found reversible two months after treatment. We consider that a time of one to two-months after laser treatment is long enough for reversibility to occur, given that the lifespan of adult *G. fossarum* in temperate rivers of comparable mean temperature (9–11 °C) is estimated at eight to 10 months (Pöckl et al., 2003), and that *P. minutus* takes at least two months to reach the cystacanth stage (Hynes and Nicholas, 1957). In addition, this long period after laser exposure should have allowed gammarids to recover from any stressful effect of laser exposure alone. Thus, at least one trait involved in increased trophic transmission of *P. minutus*, geotaxis (Jacquin et al., 2014), is irreversibly altered, independently of cystacanth viability. Decreased locomotor activity and metabolic rate were also not reversed one month after laser treatment, but this trend was no more significant for RMR two months after treatment. Irreversibility of RMR on the longer term cannot therefore be fully established.

Host recovery from parasitic manipulation has been previously reported for only three parasites with simple life-cycle, that modify their hostʼs behavior to gain protection during their development (Eberhard, 2010; Dheilly et al., 2015), or to reach a mating place once attaining sexual maturity (Ponton et al., 2011). However, to the best of our knowledge, no case of irreversible parasite-induced behavioral alteration has been reported so far. Irreversibility could possibly be the consequence of two features of our model system, a complex life-cycle with behavioral alterations involved in trophic transmission from an intermediate to a definitive host, and a long larval development time in the intermediate host. Trophically-transmitted helminth parasites undergo long larval developmental time in intermediate hosts before reaching the stage infectious to the next host, at which stage growth is stopped. According to theoretical evidence, stopping larval growth upon reaching infectivity is adaptive for helminth parasites with trophic transmission, because it would increase parasite survival by avoiding increasing time- or size-dependent host mortality (Parker et al., 2009; Chubb et al., 2010). Results from the present study suggest that growth arrest could be functionally associated with the irreversibility of behavioral manipulation for predation enhancement. This hypothesis could easily be tested by comparing other host-parasite systems with trophic transmission, in which parasites stop their growth on reaching the infective stage, such as trematodes or cestodes, or not, such as nematodes.

Contrary to behavioral changes, hemolymph total phenoloxidase activity in successfully laser-treated individuals (i.e. hosting a dead cystacanth) went back to the level observed in uninfected gammarids. The alteration of this component of immune system in *P. minutus* infected gammarid thus appears to be reversible. The elevated total phenoloxidase activity in gammarids infected with a live cystacanth contradicts a previous record on the same host-parasite species from the same population, reporting decreased ProPO-PO and hemocytes concentration in the hemolymph of gammarids infected with *P. minutus* (Cornet et al., 2009). Such discrepancy is unlikely to be related to methodological issues. First, the total phenoloxidase activity in the hemolymph of uninfected *G. fossarum* was comparable between the two studies. Secondly, the higher total phenoloxidase activity in gammarids infected with a live parasite was significant even after controlling for total protein content in the hemolymph. Increased total phenoloxidase activity in *G. fossarum* infected with live *P. minutus* could be interpreted by taking into account the origin of the outer envelope surrounding and protecting the cystacanth on the one hand, and the dynamic feature brought about by the prolonged maintenance in the laboratory and consequent cystacanth ageing on the other hand. The cystacanth of *P. minutus* is surrounded by an acellular envelop, the origin and composition of which are still debated but possibly involve membranous material of parasite origin together with residues of disintegrated hemocytes, thereby masking parasite antigens (Taraschewski, 2000; reviewed in Perrot-Minnot et al., 2023). Given that arthropod pro-phenoloxidases lack a peptide signal for their secretion, the presence of ProPO in hemolymph probably results from hemocyte lysis (González-Santoyo and Córdoba-Aguilar, 2012). Elevated ProPO levels in gammarids infected with a live cystacanth could therefore result from long-lasting permanent challenge imposed by a live parasite throughout its development, leading to regular hemocyte lysis. Given the role of ProPO in regulating hematopoiesis (Liu et al., 2021), it would in turn enhance hematopoiesis in gammarids infected with ageing cystacanth, and consecutively maintain elevated ProPO in hemolymph. Under this hypothesis, immunosuppression in the form of depressed ProPO and hemocyte concentrations (as evidenced by Cornet et al., 2009) could be a transitory step in gammarids infected with younger cystacanths. This interpretation is compatible with the return of ProPO concentration to “normal” level once the parasite does not stimulate hematopoiesis and hemocyte lysis anymore. Interestingly, such a defensive strategy would allow the parasite to avoid paying the cost of maintaining resistance against host immune responses (Chubb et al., 2010). In addition, we observed that dead cystacanth were not encapsulated up to two months after death. Most probably, as the envelop was not damaged by the laser treatment (Fig. 1), dead cystacanth was not recognized as non-self even in the absence of an active defense from the parasite. This interpretation is supported by the comparable ability of gammarids to encapsulate an inorganic immunogen independently of infection status, thereby bringing evidence for a “masking property” of the parasiteʼs outer envelope. Detailed histological and cytological studies are needed to test the hypothesis of stimulated hematopoiesis and hemocytes lysis, and to confirm this protective “masking” effect of the outer envelope. In addition, testing the “altered hematopoiesis and hemocyte lysis” hypothesis, possibly accounting for increased PPO with ageing parasite and dead parasite protection against melanotic encapsulation, requires the comparison of hemocyte concentration between gammarids infected with a live parasite (both unexposed or having survived laser treatment) and gammarids hosting a laser-killed parasite. Finally, a complementary and important component of innate immunity in crustaceans should be investigated in this host-parasite system, the antimicrobial peptides (AMPs) (Matos and Rosa, 2022). Although there is yet no report on the impact of acanthocephalan infection on AMPs release, investigating several immune effectors together would provide a better understanding of gammarid-*P. minutus* interaction than considering only the phenoloxidase system.

At the proximate level, the increased brain lactate in gammarids infected with a live cystacanth returned to “normal” in gammarids carrying a dead cystacanth, two months after parasite death. The reversibility of brain lactate level is unlikely to be a consequence of the concomitant recovery of RMR following parasiteʼs death, because such increase in RMR also occurred in gammarids infected with a live parasite after two months of maintenance, whereas their level of brain lactate remained high. The reversibility of elevated brain lactate and irreversibility of geotaxis following parasite death may challenge at first sight the previous proposition that lactate would trigger altered geotaxis (Perrot-Minnot et al., 2016). Following Perrot-Minnot et al. (2016), the pathways involved in anaerobic metabolism and hypoxia signaling could be responsible for the changes in geotaxis and metabolic rate associated with *P. minutus* infection. The underlying physiological mechanism could correspond to the excretion-secretion by the cystacanth of lactate, or of a hypoxia-signaling factor (HIF) triggering elevated brain lactate (Perrot-Minnot et al., 2016). Alternatively, elevated brain lactate could be of host origin, as a consequence of altered neuroenergetics in infected hosts. Interestingly, however, elevated brain lactate level may not be necessary to maintain associated phenotypic alterations. Indeed, recent studies have revealed that lactate mediates epigenetic changes in mammals, more specifically histone lactylation (Xie et al., 2022; Li et al., 2023). If epigenetic changes are at play, a permanent increase in brain lactate level might not be necessary to maintain associated phenotypic alterations, as evidenced here in gammarids hosting a dead parasite. The full assessment of the cost-effectiveness of behavioral manipulation therefore requires further research on lactate signaling functions and neuroepigenetics in the crustacean brain. Investigations on the role that parasite-mediated epigenetic changes could play in host manipulation has been limited so far to maternal effects and multidimensionality of parasite-induced alterations in another trophically-transmitted parasite, the protozoan *Toxoplasma gondii* (Pavey and Vyas, 2023). Yet, the “extended epiphenotype” (Pavey and Vyas, 2023) is a sound hypothesis to explain the irreversibility of parasite-induced behavioral alterations on other host-parasite systems, as reported here. In that respect, progress in methodology allowing the neutralization of manipulative parasites without affecting their intermediate hosts may open new avenues for research on the causes and consequences of host manipulation by parasites. Importantly, our study illustrates the information gained and new hypothesis raised by addressing manipulation reversibility.

## 5 Conclusions

Evidence for partial reversibility of phenotypic alterations in gammarids infected with acanthocephalans opens a new horizon for the study of host manipulation by parasites. The next challenge consists of developing tools allowing the neutralization of macroparasites inside their hosts in a wide range of host-parasite systems. Indeed, comparative evidence on the reversibility of parasite-induced phenotypic alterations should improve our understanding of both the ultimate mechanisms of host manipulation, particularly its associated physiological costs, and its proximate mechanisms, including the putative role of epigenetic alterations and the ways parasite larvae can overcome and evade host immune defenses.

## CRediT authorship contribution statement

**Marie-Jeanne Perrot-Minnot:** Conceptualization, Methodology, Validation, Investigation, Formal analysis, Data curation, Writing – original draft, Writing – review & editing, Visualization. **Frank Cézilly:** Conceptualization, Formal analysis, Writing – original draft, Writing – review & editing. **Olivier Musset:** Conceptualization, Methodology, Resources, Software, Validation, Investigation, Writing – original draft, Writing – review & editing, Visualization, Funding acquisition.

## Ethical approval

The study complies with the rules of ethics on crustaceans, as prescribed by the French legislation and the Université de Bourgogne.

## Funding

This research was funded by grants from Région Bourgogne Franche-Comté and Feder (PARI, 2016-9201AAO050S01787 and FEDER BG0005895).

## Declaration of competing interests

The authors declare that they have no known competing financial interests or personal relationships that could have appeared to influence the work reported in this paper.

## Data Availability

The data supporting the conclusions of this article are included within the article and its supplementary file. Raw data are deposited in Mendeley Data, V1, doi: 10.17632/gy6xh94yjp.1.

## Appendix A Supplementary data

Supplementary file S1**.**Supplementary material S1**.** Geotaxis one and two months after laser treatment, and behaviural repeatability. Supplementary material S2. Dry body weight of gammarids maintained in the lab for one month or two months, and used to record resting metabolic rate. Supplementary material S3. Locomotor activity: detailed method and additional figures. Supplementary material S4. Total phenoloxidase activity and total protein concentration in hemolymph of *G. fossarum* according to infection status and *P. minutus* viability. Supplementary material S5. Testing for the ability of gammarids harboring a dead parasite to encapsulate an inorganic and non-pathogenic foreign body: immune response to an implanted nylon monofilament. Supplementary material S6**.** Effect of laser exposure *per se* on gammarids: geotaxis, RMR and brain lactate concentration of gammarids infected with a parasite having survived laser treatment.
