# Supplementary material for: Laser-based selective killing of a manipulative parasite reveals partial reversibility of phenotypic alterations in its intermediate host
Source: Curr Res Parasitol Vector Borne Dis. 2024 Oct 11;6:100221. doi: 10.1016/j.crpvbd.2024.100221 (PMC11647135; doi:10.1016/j.crpvbd.2024.100221)
Supplement: Multimedia component 1 [file mmc1.docx]

Laser-based selective killing of a manipulative parasite reveals partial reversibility of phenotypic alterations in its intermediate host

Electronic supplementary material

**Supplementary material S1.** Geotaxis one and two months after laser treatment, and behavioral repeatability.

**Supplementary material S2.** Dry body weight of gammarids maintained in the laboratory for one month or two months, and used to record resting metabolic rate.

**Supplementary material S3.** Locomotor activity: detailed method and additional figures.

**Supplementary material S4.** Total phenoloxidase activity and total protein concentration in hemolymph of *G. fossarum* according to infection status and *P. minutus* viability.

**Supplementary material S5.** Testing for the ability of gammarids harboring a dead parasite to encapsulate an inorganic and non-pathogenic foreign body: immune response to an implanted nylon monofilament.

**Supplementary material S1. Geotaxis behaviour one and two months after laser treatment, and behavioral repeatability.**

There was no difference in geotaxis one or two months after laser treatment and maintenance under controlled laboratory conditions in any of the four groups (Wilcoxon Mann-Whitney test: infected controls, W = 1270, *p* = 0.23; gammarids infected with *P. minutus* having survived to laser treatment, W = 84, *p* = 0.52; gammarids infected with laser-killed *P. minutus*, W = 1043, *p* = 0.06, uninfected gammarids, W = 1711, *p* = 0.25; Supplementary Figure S1). In addition, geotaxis was significantly repeatable in all four gammarid groups (Spearman rank correlation: infected controls: N = 78, *r*_s_ = 0.4, *p* = 0.0003; gammarids infected with *P. minutus* having survived to laser treatment: N = 19, *r*_s_ = 0.62, *p* = 0.004; gammarids infected with laser-killed *P. minutus*: N = 60, *r*_s_ = 0.27, *p* = 0.04; uninfected: N = 90, *r*_s_= 0.3, *p* = 0.004).


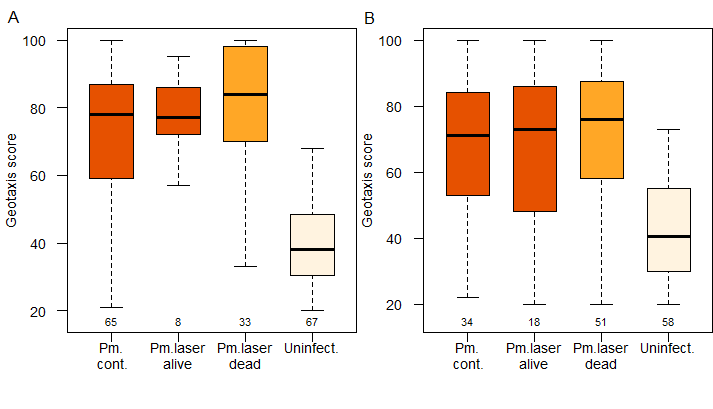


**Supplementary Figure S1.** Geotaxis of *G. fossarum* individuals harbouring a live *P. minutus* cystacanth not exposed to laser (Pm.cont) or having survived to laser treatment (Pm.laser alive), individuals harbouring a cystacanth killed by laser treatment (Pm.dead), and uninfected ones (Uninfect.). The scores were recorded one month (A) or two months (B) after laser-based killing of the parasite and/or maintenance in the lab. Sample size is given below bars.

**Supplementary material S2. Dry body weight of gammarids used in the recording of geotaxis, locomotor activity (one month only), and resting metabolic rate.**

Dry body weight of gammarids was comparable across gammarid groups (X² = 0.63, df = 3, *p* = 0.89), but increased significantly between one month and two months maintenance in the laboratory (glm, gaussian family and log link function, reduced model: X²= 14.92, df = 1, *p* = 0.0001; R² = 0.05; Supplementary Figure S2). Gammarid dry weight was slightly higher after two months of maintenance in the laboratory under controlled photoperiod and temperature conditions (12:12 D/L; 16 °C).


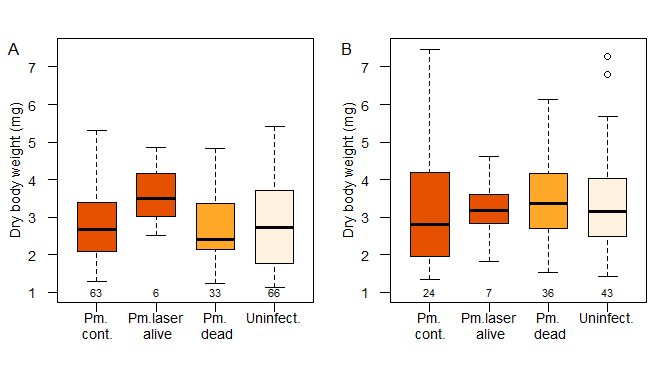


**Supplementary Figure S2.** Dry body weight of *G. fossarum* infected with live *P. minutus*, either not exposed to laser treatment (Pm.cont.) or having survived to laser treatment (Pm.laser alive), or with laser-killed *P. minutus* (Pm.dead), and of uninfected gammarids (Uninfect.), after one month (A) and two months (B) maintenance in the lab, following treatment. Sample size is given below bars.

**Supplementary material S3. Locomotor activity: proportion of time swimming**


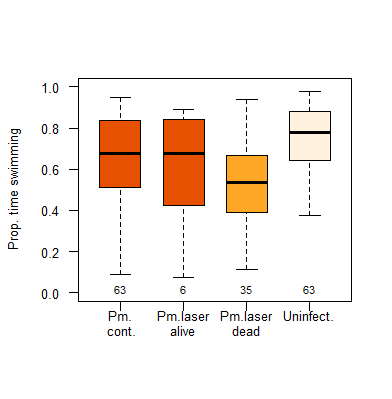


**Supplementary Figure S3.** Swimming activity of *G. fossarum* infected with *P. minutus* or uninfected, recorded one month after laser-based killing of the parasite as the proportion of time spent moving above the speed threshold of 15 mm.s^-1^). Gammarids were infected with either a live cystacanth not exposed to laser (Pm.cont.), a cystacanth having survived to laser treatment (Pm. laser alive), a dead cystacanth of *P. minutus* (Pm.dead), or were uninfected (Uninfect.).

Sample size is given below bars.

**Supplementary material S4. Hemoplymph PPO-PO and total protein concentration**

A B


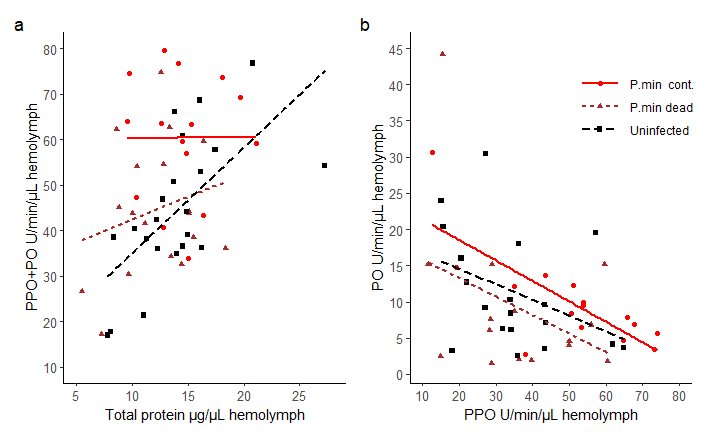


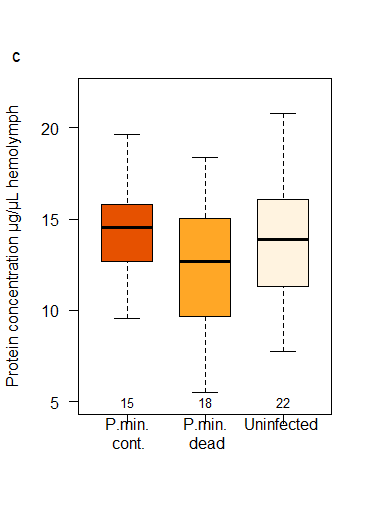


**Supplementary Figure S4.** Total phenoloxidase activity and protein concentration in the hemolymph of *G. fossarum* according to infection and parasite viability: infected with live *P. minutus* (P.min control, A) or laser-killed *P. minutus* (P.min dead; B), or uninfected (black; C). (A) Total phenoloxidase activity (PPO+PO) regressed on hemolymph total protein concentration; (B) Protein concentration in hemolymph.

**Supplementary material S5. Testing for the ability of gammarids harboring a dead parasite to encapsulate an inorganic and non-pathogenic foreign body: immune response to an implanted nylon monofilament.**

To assess the capacity of gammarids harboring a dead cystacanth to encapsulate a non-self body, we challenged their immune system with an inorganic and non-pathogenic immunogen (Armitage et al., 2003). To that end, we implanted a 1-mm nylon monofilament in gammarids, following exposure to laser treatment and six-week maintenance in the lab, using the same protocol and population as the ones used to perform behavioral assay and physiological measurements (see Material and methods section) and a separate batch of individuals (collected in Feb. 2024 and randomly allocated to each experimental group). Sample size for infected gammarids exposed to laser treatment dropped however to one-fourth of all gammarids exposed, due to unexpected mortality in this group only after six-week maintenance. Implants were cut in a nylon (fishing) wire of 0.15 mm diameter, previously rubbed with sandpaper to increase roughness and soaked into crustacean saline solution until use. The gammarids were first anesthesized in MS-222 following Perrot-Minnot et al. (2021) and immobilized on a piece of clay before inserting the nylon implant. We gently punctured the cuticle laterally at the level of the third or fourth coxal plate with a sharpened steel wire, and inserted the implant inserted up to 2/3 of its length using fine forceps. Gammarids were then returned to maintenance boxes for six days. After six days, we anesthetized the gammarids, measured their body size (height of the fourth coxal plate), and the length of the inserted part of the nylon filament after transferring it into a drop of glycerol, under a stereoscopic microscope Nikon SMZ-1500. We quantified the level of melanization from pictures taken at 40x magnification, using Image J software v. 1.54g to estimate integrated density (sum of pixels) (Schneider et al., 2012). We took four measurements of the inserted length of nylon implant and four pictures for melanization quantification per implant /gammarid, and used averaged values for statistical analysis. We analyzed the density of pigmentation according to gammarids group, gammarid size and implant surface, using linear model. We run an analysis of deviance and tested for the effect of each predictor using the sums of squares for each predictor conditional on the other predictors and associated P-values (type II tests) (R-package “car” v.3.1-2, Fox and Weisberg, 2019). We run a power analysis a posteriori using package “pwr” (v.1.3-0, Champely, 2020).

The density of melanization was influenced by implant surface (χ² = 7.54, d.f. = 1, *p =* 0.006), but not gammarid status χ² = 0.82, d.f. = 2, *p =* 0.66) nor size (χ² = 1.75, d.f. = 1, *p =* 0.19; R² = 0.18, power = 0.85). The ratio of melanization density to implant surface was not different among gammarid groups (Supplementary Figure S5). There was no significant difference between groups in the size of gammarids or the implant surface (linear models: F_2,52_ = 1.18, *p =* 0.32 and F_2,52_ = 0.42, *p =* 0.66, respectively).

**
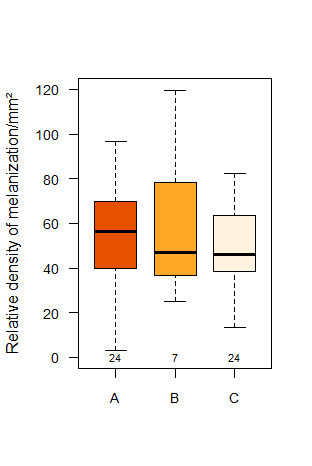
**

**
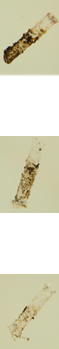
**

**Supplementaty Figure S5.** Ability of gammarids to encapsulate an inorganic and non-pathogenic nylon filament, estimated as the density of pigmentation relative to nylon implant surface (ratio), according to infection status: gammarids infected with *P. minutus* not exposed to laser (A) or laser-killed (B), or uninfected (C).

**References**

Armitage SAO, Thompson JJW, Rolff J, Siva-Jothy MT 2003. Examining costs of induced and constitutive immune investment in Tenebrio molitor. *J. Evol. Biol.* **16**, 1038–1044.

Champely S (2020). pwr: Basic functions for power analysis_. R package version 1.3-0, https://CRAN.R-project.org/package=pwr.

Perrot-Minnot M-J, Balourdet A, Musset O 2021. Optimization of anesthetic procedure in crustaceans: evidence for sedative and analgesic-like effect of MS-222 using a semi-automated device for exposure to noxious stimulus. *Aquat. Toxicol*. **240**, 105981.

Schneider CA, Rasband WS, Eliceiri K. W 2012. NIH Image to ImageJ: 25 years of image analysis. *Nat. Methods.* **9**, 671–675.
